# A case of pericardial tuberculoma presenting as a left anterior chest wall mass

**DOI:** 10.1186/1755-7682-6-48

**Published:** 2013-12-19

**Authors:** Mohamed Leye, Modou Jobe, Souleymane Diatta, Mouhamadou Bamba Ndiaye, Fatou Aw, Gabriel Nounignon Comlan Deguenonvo, Alain Affangla, Pape Souleymane Toure, Madoky Magatte Diop, David Messika Zeitoun

**Affiliations:** 1 Service de Cardiologie, Centre Hospitalier Universitaire de Fann, Dakar, Sénégal; 2 Unité de formation et de recherches des sciences de la santé, Université de Thiés, Thiés, Sénégal; 3 Service de cardiologie, CHU Aristide Le Dantec, Dakar, Sénégal; 4 Service de chirurgie thoracique et cardiovasculaire du CHU de Fann, Dakar, Sénégal; 5 Service de cardiologie, CHU Bichat, Paris, France

**Keywords:** Pericardial tuberculoma, Chest wall mass, Tuberculosis, Senegal

## Abstract

**Introduction:**

Tuberculosis (TB) can present both in its pulmonary or extra-pulmonary forms. Cardiac tuberculoma previously described only after autopsy is continuously seen with the advent of more advanced imaging modalities.

**Case report:**

A 23-year-old male with a four month history of a progressively increasing left anterior thoracic wall mass of 5 cm in diameter was referred from oncology for clinical re-evaluation and for echocardiography. Systemic examination was essentially normal. Transthoracic and trans-oesophageal echocardiography showed the presence of a pericardial mass around the right atrioventricular junction. Thoracic CT scan showed an anterior mass in left chest wall extending to the pericardium and also the presence of mediastinal lymphadenopathy. Mantoux test was positive and histological examination of tissue biopsy confirmed the presence of TB. However, blood tests and culture of aspirated purulent fluid were unyielding. A diagnosis of pericardial tuberculoma with mediastinal and parietal extension was made and patient was successfully treated with standard anti-TB chemotherapy.

**Discussion:**

The possible differential diagnoses for chest wall tumors are varied and a high degree of suspicion is needed to diagnose cardiac tuberculoma especially in endemic regions. Imaging though helpful in morphological description cannot make precise diagnosis. The diagnosis depends on histological and culture studies. There is usually a good evolution with anti-TB treatment.

**Conclusion:**

In an era of an increasing number of acquired immune-compromised patients, and with increasing number of diagnoses of tuberculosis, a diagnosis of cardiac tuberculoma should be considered in patients presenting with a thoracic wall mass.

## Introduction

Tuberculosis (TB) is a global health concern despite the widespread attention that it has received over the years. Pulmonary tuberculosis is the most commonly recognized form, however, extra-pulmonary manifestations of tuberculosis account for about 15-20% of cases [1,2]. Tuberculous pericarditis, caused by Mycobacterium tuberculosis, is found in approximately 1% of autopsy cases and in 1% to 2% of cases of pulmonary TB [3]. It is the most common cause of pericarditis in Africa and in other developing countries in which TB remains a major public health problem [4]. Cardiac tuberculoma previously described only after autopsy is continuously seen with the advent of more advanced imaging modalities [5].

## Case report

**Figure 1:**
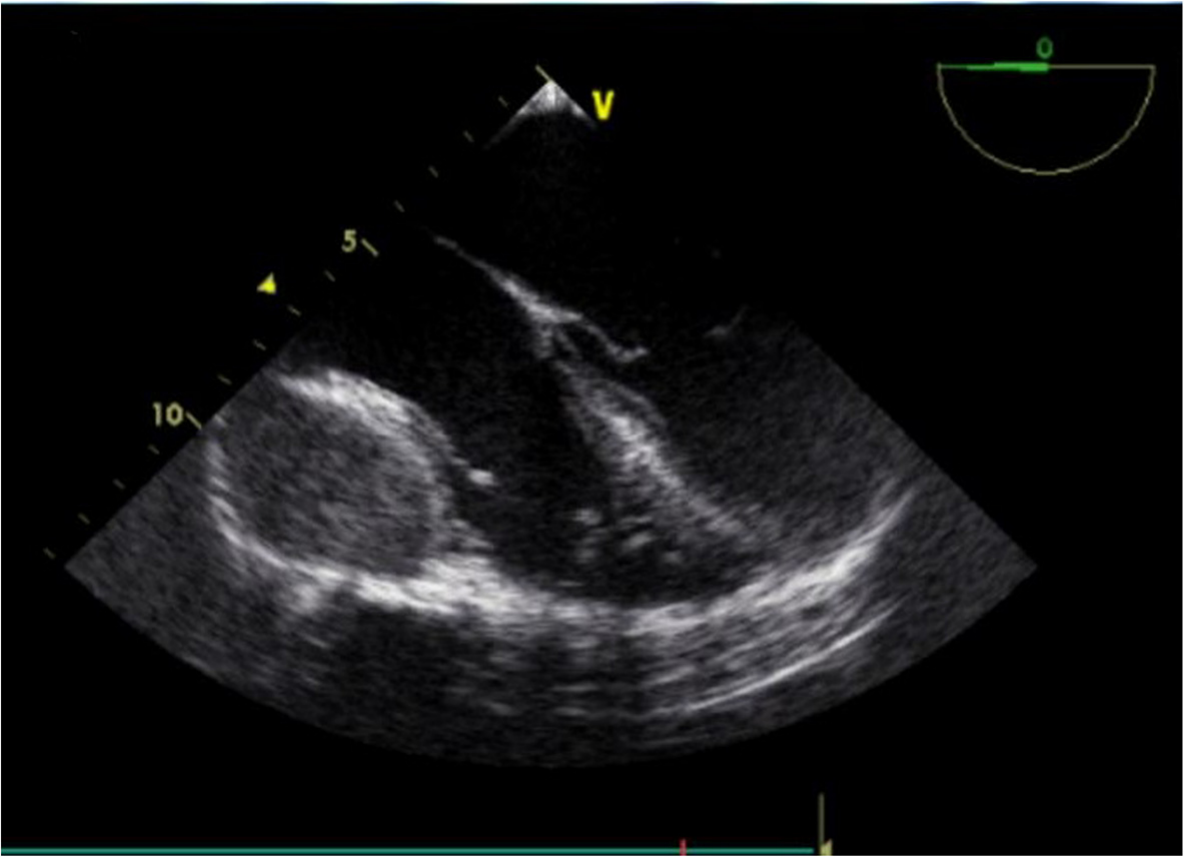
Bidimensionnal multiplane transoesophageal echocardiography: apical 4-chamber view incidence 0° showing mass in the pericarial mass around the right auriculo-ventricular junction.

**Figure 2:**
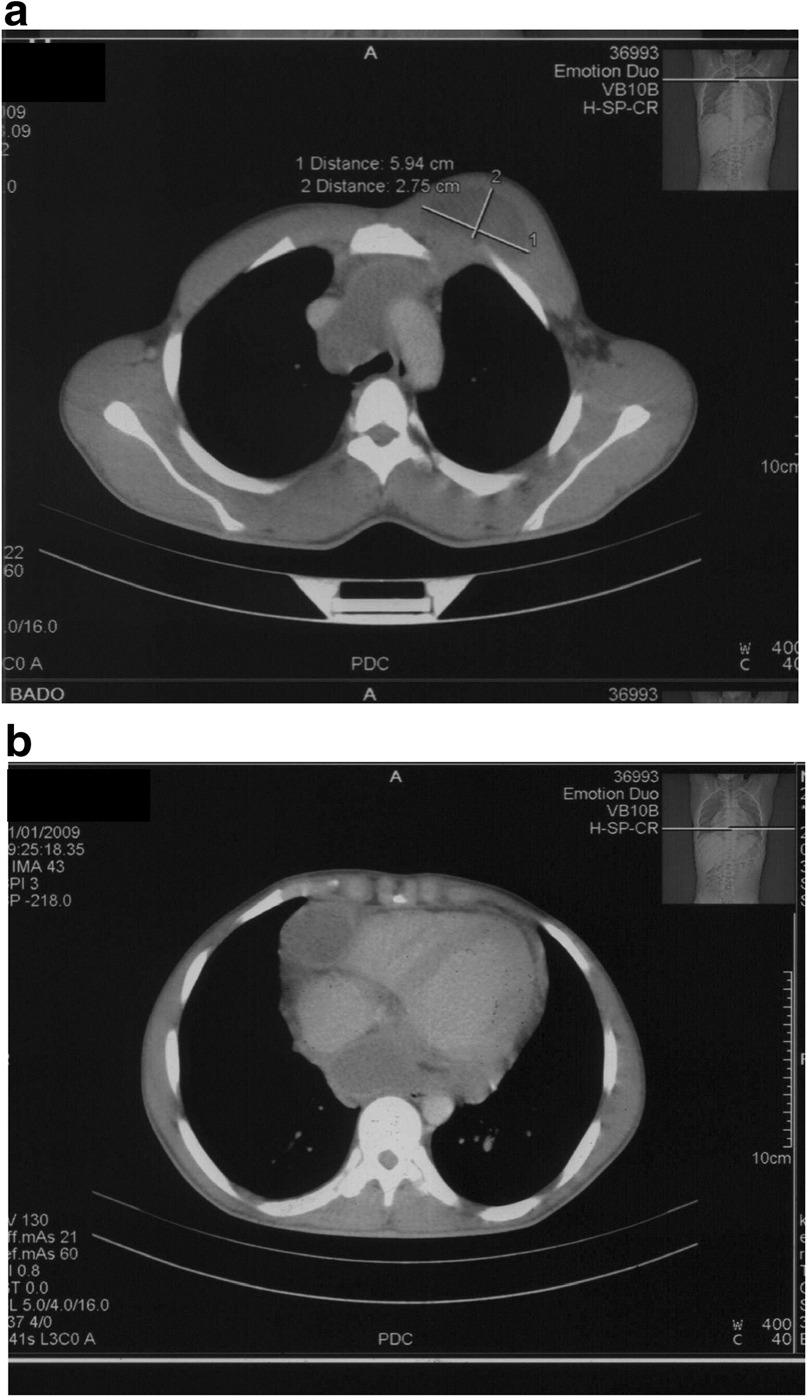
**Chest CT scan at presentation. a.** Mass visualized in left anterior chest wall measuring 5.9 cm×2.7 cm. **b.** Pericardial mass visualized around the right atrioventricular junction.

**Figure 3:**
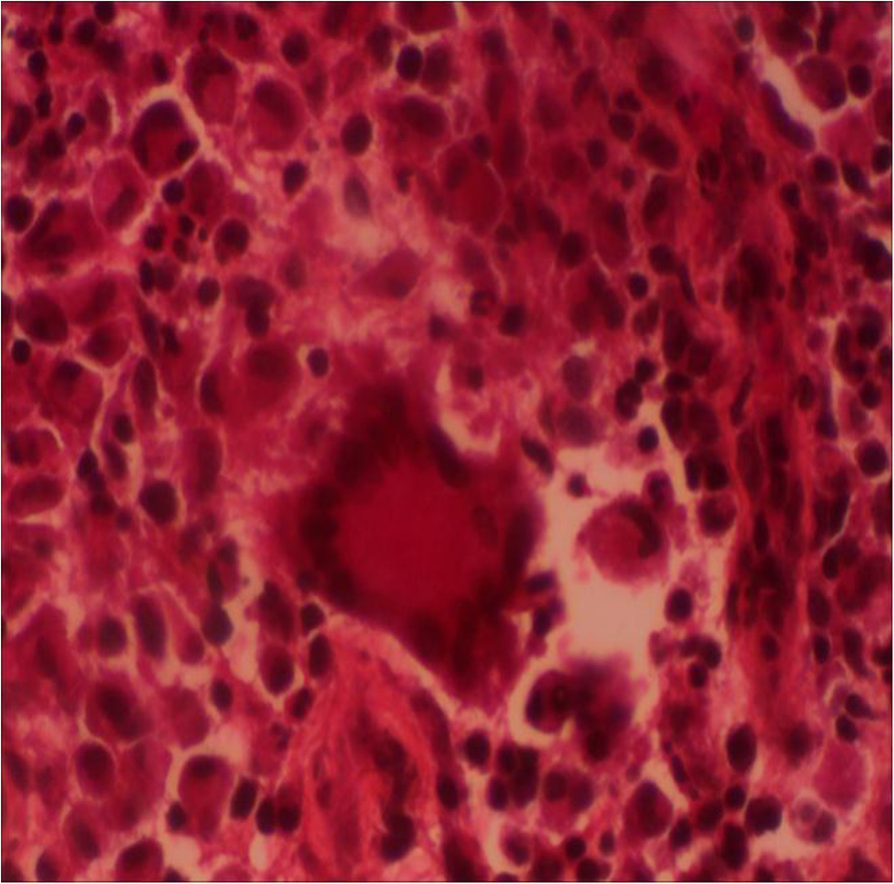
**Photomicrograph of the tissue biopsy showing a Langhans type giant cell (with nuclei arranged in a ring at the periphery) surrounded by epithelial cells and lymphocytes.** Tuberculous granuloma.

A 23-year-old male with an irrelevant past history presented with a four month history of a progressively increasing left anterior thoracic wall mass of 5 cm in diameter. With this, he initially had a cardiology consultation and after clinical evaluation was referred for an oncology review and management for a suspicion of a malignancy. Before any intervention at the latter department, he was again referred to cardiology for re-evaluation and for echocardiography. Systemic examination was essentially normal. Transthoracic and trans-oesophageal echocardiography (Figure 1) were done and both showed the presence of a pericardial mass around the right atrioventricular junction. There was no compression of cardiac structures. The transmitral velocity profile was normal with no elevation of filling pressures. Further assessment with a thoracic CT scan showed an anterior mass in the left chest wall extending to the pericardium (Figure 2) and also noted the presence of mediastinal lymphadenopathy. Blood tests including an HIV test were unyielding but however, Mantoux test was positive, and histological examination of tissue biopsy showed multiple inflammatory granulomas with lymphocytes, epithelial cells and Langhans giant cells consistent with the presence of TB (Figure 3). However, culture of aspirated purulent fluid was unyielding. A diagnosis of pericardial tuberculoma with mediastinal and parietal extension was made. He was successfully treated with standard 9-month anti-TB chemotherapy. Six months after starting the aforementioned treatment there was a complete disappearance of the mass confirmed by echocardiography with no sign of constrictive pericarditis.

## Discussion

Cardiac tuberculosis was first reported by Maurocordat in 1664 and by Morgagni in 1761 [6]. Cardiac tuberculomas can affect all four chambers of the heart, and simultaneous manifestations of tuberculomas at different locations have been reported. The right heart is mostly affected probably because of the frequent involvement of the right mediastinal lymph nodes with consequent spread to the myocardium [7].

The differential diagnosis of masses of the anterior and mid-mediastinum [8] includes thymomas, lymphomas, teratomatous neoplasms, thyroid masses, vascular masses, lymph node enlargement due to metastases or granulomatous disease, and pleuropericardial and bronchogenic cysts. Whereas the diagnosis of cardiac tuberculoma was almost exclusively made at autopsy previously, advances in imaging techniques give an opportunity for earlier diagnosis. These advanced imaging techniques may contribute to an optimal morphologic description, assessment of hemodynamic significance as well as their surveillance [5]. However, they cannot help in distinguishing cardiac tuberculomas from other tumors. Generally, tissue diagnosis by endomyocardial biopsy and Ziehl-Neelsen staining are the most specific tests. However, this often fails to reveal the acid fast bacilli, and a definitive diagnosis depends on seeing typical histological changes. A culture of the organism can be done for definitive diagnosis.

Complete regression of tuberculoma treated with standard anti-TB chemotherapy has been reported [5] as in our case. Surgical intervention may be required in large tuberculoma when pharmacotherapy alone is inadequate and in cases of severe hemodynamic compromise, threatening thromboembolism or refractory arrhythmias [9].

## Conclusions

We presented in this case a rare form of extra pulmonary TB. To our knowledge this is the first reported case in the literature in an immunocompetent patient with TB presenting as an anterior chest wall mass. A high index of suspicion is needed for its diagnosis especially in highly endemic regions as patients can be successfully treated with standard anti-TB chemotherapy. In an era of increasing number of acquired immuno-deficiency syndrome patients, and with increasing number of diagnoses of tuberculosis, a diagnosis of cardiac tuberculoma after a thorough clinical evaluation should be considered in patients presenting with a thoracic wall mass.

## Consent

Written informed consent was obtained from the patient for publication of this Case report and any accompanying images. A copy of the written consent is available for review by the Editor-in-Chief of this journal.

## Competing interests

The authors declare that they have no competing interest.

## Authors’ contribution

ML and MJ conducted the literature search, drafted the first manuscript, performed language correction, and participated in article design and coordination. ML and MBN conducted the echocardiography and participated in manuscript draft. GNCD, SD, FA and DMZ critically revised the manuscript for important intellectual content. AA, PST and MMD participated in investigation studies and critically evaluated the article. All authors read and approved the final manuscript.

## References

[B1] Batra R Trehan V Salwan R Krishan A Nigam M Malhotra V Kaul UA Arora R Antemortem diagnosis of cardiac tuberculoma Indian Heart J 1998 6 1 87 89 9583298

[B2] Fanning A Tuberculosis: extra pulmonary disease CMAJ 1999 6 1597 1603 10374005 PMC1230370

[B3] Fowler NO Tuberculous pericarditis JAMA 1991 6 99 103 10.1001/jama.1991.03470010103039 2046135

[B4] Mayosi BM Volmink JA Commerford PJ Yusuf S, Cairns JA, Camm AJ, Fallen BJ Pericardial disease: an evidence-based approach to diagnosis and treatment Evidence-based cardiology 2003 2 London: eBMJ Books 735 748

[B5] Mayosi BM Burgess LJ Doubell AF Tuberculous pericarditis Circulation 2005 6 3608 3616 10.1161/CIRCULATIONAHA.105.543066 16330703

[B6] Njovane X Intramyocardial tuberculosis- a rare underdiagnosed entity SAMJ 2009 6 153 153 19563088

[B7] Jeilan M Schmitt M McCann G Davies J Leverment J Chin D Cardiac tuberculoma Circulation 2008 6 984 986 10.1161/CIRCULATIONAHA.107.691766 18285579

[B8] Cantinotti M De Gaudio M de Martino M Assanta N Moschetti R Veneruso G Crocetti M Murzi B Chiappini E Galli L Intracardiac left atrial tuberculoma in an eleven month- old infant: case report BMC Infect Dis 2011 6 359 10.1186/1471-2334-11-359 22208878 PMC3268750

[B9] Jouannic I Pavin D Seguin P Arvieux C Paumier V Camus C Le Tulzo Y Leguerrier A de Place C Thomas R Tuberculome cardiaque; intérêt de l’échographie cardiaque et discussion thérapeutique à propos d’un cas Arch Mal Coeur Vaiss 1995 6 3 401 404 7487295

